# Adolescent and covert family planning users’ experiences self-injecting contraception in Uganda and Malawi: implications for waste disposal of subcutaneous depot medroxyprogesterone acetate

**DOI:** 10.1186/s12978-020-00964-1

**Published:** 2020-08-03

**Authors:** Holly M. Burke, Catherine Packer, Laura Wando, Symon Peter Wandiembe, Nelson Muwereza, Subarna Pradhan, Akuzike Zingani, Bagrey Ngwira

**Affiliations:** 1 Reproductive, Maternal, Newborn and Child Health, FHI 360, 359 Blackwell Street, Suite 200, Durham, North Carolina 27701 USA; 2 WellShare International, PO Box 35514, Kampala, Uganda; 3 N&E Consult International Limited, PO Box 75686, Makerere Hill Kampala, Uganda; 4 University of Malawi, Polytechnic, PO Box 303, Chichiri, Blantyre 3, Malawi

**Keywords:** Adolescent, Discreet, Covert, Waste, Disposal, Self-injection, Self-administer, Subcutaneous depot medroxyprogesterone acetate, DMPA-SC, Uganda, Malawi, Community health worker

## Abstract

**Background:**

Self-administered subcutaneous depot medroxyprogesterone acetate (DMPA-SC) is poised to increase access to contraception; however, governments are concerned about the waste management of used units. Self-injectors in Malawi and Uganda are currently instructed to store used units in containers and return them to health workers for disposal. However, this may not be feasible in low-resource settings, especially for younger or covert self-injectors. We describe adolescent (15–19 years) and adult (20–49 years) self-injectors’ disposal experiences in Uganda and Malawi. When possible, we compare covert and overt users’ experiences.

**Methods:**

We conducted cross-sectional qualitative studies in 2019 with 50 self-injectors in Uganda and 60 in Malawi. We purposively selected approximately half adolescents and included those trained by clinic-based providers and community health workers. We conducted semi-structured interviews and thematic data analysis and compared the findings across settings.

**Results:**

Just under half of both samples were adolescents, substantially more of whom were covert users in Uganda (68%) than Malawi (~ 10%). Most participants reported being told to store used units in a container and return them to health workers. About two-thirds of Uganda participants had disposed of at least one unit by the interview, most commonly returning them to health workers. Over one-third of Malawi participants had disposed of at least one unit by the interview, slightly more disposed into latrines compared to returning to health workers. Participants in both settings reported compliance with health workers’ disposal instructions as a primary reason for their disposal method. One-fifth of Uganda participants, mostly adolescent covert users, and one-quarter in Malawi said they were told they could dispose into latrines, and often did so. The majority in both settings said they would prefer to dispose units in latrines because they worried about needlestick injuries to others and because it was convenient. Some Uganda adolescent covert users felt returning units to health workers was challenging due to privacy concerns.

**Conclusions:**

While most self-injectors disposed of used units as instructed, findings from both studies suggest that returning units to health workers is not preferred and may not be feasible for some adolescent covert users. More convenient disposal solutions should be identified.

## Plain English summary

Adolescents, and those who covertly use family planning without their partners or families knowing, face barriers when using injectable contraceptives. These groups may benefit from giving themselves injections in home settings (“self-injection”), instead of receiving injections from health workers. Subcutaneous depot medroxyprogesterone acetate (DMPA-SC) is a self-injectable contraceptive. Each prefilled DMPA-SC unit provides 3 months of pregnancy protection. Governments are concerned about how self-injectors discard used units. Self-injectors in Uganda and Malawi are asked to store used units in containers and return them to health workers at their convenience. Health workers then discard the units into medical waste containers. However, this disposal procedure may not work well for adolescents and covert self-injectors because of privacy concerns. In 2019 we interviewed 50 women and girls in Uganda, and 60 in Malawi, to hear about their experiences disposing units. We interviewed adolescents (15–19 years) and adults (20–49 years), and when possible, covert self-injectors. Participants mainly disposed of units in the way health workers told them to. While many returned units to health workers, some were told they could dispose into the latrine and did so. Most said they would prefer to dispose of units in latrines because they worried about needlestick injuries to others and because it was convenient. Findings from both studies suggest that returning units to providers is not preferred and may not be feasible for some adolescent covert users. More convenient disposal solutions should be identified. Based on our findings, we offer recommendations for family planning programs that include self-injected DMPA-SC.

## Background

Early childbearing is common in Uganda and Malawi, and there is high unmet need for family planning among adolescents ages 15–19 years in both countries [1, 2]. The modern contraceptive prevalence rate (mCPR) of married adolescents ages 15–19 years is low at 21% in Uganda and 38% in Malawi, while for unmarried adolescents in the same age group, the mCPR is 43% in Uganda and 52% Malawi [1, 2]. In both countries, injectable contraception is the most common method, used by 19% of married women ages 15–49 years in Uganda and 30% in Malawi [1, 2].

Contraceptive preferences in Uganda, Malawi, and elsewhere in sub-Saharan Africa vary according to user population. Adolescents, especially those who are not married or have not begun childbearing, fear stigma from community members and health workers for seeking family planning (FP) care [3–5]. Embarrassment in seeking contraceptive services often results in adolescents seeking care from health workers offering faster, more discreet services and methods [3, 6].

Covert or discreet users (i.e., women who use FP methods unbeknownst to their partners or families) face similar confidentiality challenges, which constrain contraceptive use: disagreement between women and their partners around fertility preferences and the common belief that women using FP are promiscuous compels some women to use FP methods covertly despite partner opposition [7, 8]. Injectables have become a popular method among such women in sub-Saharan Africa because they can be administered without the partner’s knowledge [7–11]. Some adolescent FP users are also covert users which compounds their challenges using contraception. Self-administration of injectables involves teaching clients how to inject the method and giving clients additional doses to take away for future use, thereby reducing the number of visits to health workers. What is more, research on contraceptive preferences of adolescent and covert FP users suggests that self-administered injectables may appeal to both populations [12, 13]. A subcutaneous (SC) version of depot medroxyprogesterone acetate (DMPA-SC) in a prefilled, Uniject™ injection system (Sayana Press with 104 mg of medroxyprogesterone acetate in 0.65 mL suspension for injection), is an easy-to-use, self-injectable contraceptive option. Research has demonstrated DMPA-SC self-injection to be acceptable, feasible, safe and improve continuation rates [12–22], however, the need to dispose of used product after injecting themselves outside of clinical settings might present problems for adolescent and covert self-injectors.

While there is substantial global guidance on medical sharps waste management available at the facility and community levels [23], there is limited global guidance at the household level. As of 2018, the WHO included DMPA-SC in the their Essential Medicines List [24], but their guidelines [25] for health workers state that local programs should decide how to help self-injectors dispose of used needles. As governments plan for integrating DMPA-SC self-injection in their FP programs, they are concerned about the health and environmental implications of how self-injectors will dispose of the used DMPA-SC units. Recent guidance for countries developing DMPA-SC self-injection programs notes that disposal into pit latrines (convenient locations throughout sub-Saharan Africa, including in urban areas especially among low-income earners [26, 27]) is not viewed favorably by stakeholders due to unquantified environmental concerns [28, 29]. Currently in Uganda and Malawi, self-injectors are instructed to store the used units in puncture-proof containers until they can be returned to a health worker for disposal in medical sharps waste containers; however, this recommendation may not be feasible in low-resource settings, especially for younger and/or covert self-injectors. Indeed, a rapid pilot of the Uganda self-injection scale-up found that there was still a need to test locally sourced disposal methods [30].

We conducted two qualitative studies with self-injectors, including adolescents and covert users, in Uganda and Malawi—countries that had recently introduced DMPA-SC self-injection in their programs—to explore their experiences with waste disposal. Our objective was to determine whether current recommendations for waste disposal were feasible and equitable in low-resource settings where DMPA-SC and self-injection were being scaled up. These two populations of users are of particular interest given the potential appeal of the method for them paired with the associated need to dispose of the used units. Notably, this is the first study to explore waste management emphasizing the experiences of adolescent and covert self-injectors. The study findings will be used to inform service delivery in Uganda and Malawi, as well as program planning for other countries interested in integrating DMPA-SC self-injection into their FP method mix.

## Methods

### Study setting

DMPA-SC was introduced in Uganda in 2014 for administration by clinic-based providers and in communities through administration by community health workers, known as village health team members (VHTs). WellShare International built the capacity of district staff and clinic-based providers to train and supervise VHTs in Uganda to scale up community-based FP. In April 2018, WellShare first introduced DMPA-SC as a self-injectable method in six sub-counties in Iganga and Bugweri Districts (referred to as Phase 1 sites), and in August 2018, WellShare added an additional six sub-counties in Iganga and Bugweri districts (referred to as Phase 2 sites). This study took place in these 12 sub-counties. Since the time of this study, in 2019, the Uganda Ministry of Health authorized DMPA-SC self-injection nationally in both private and public sectors.

In May 2018, the Malawi Ministry of Health (MOH) Senior Management Team approved the simultaneous introduction of DMPA-SC administered by health workers and DMPA-SC for self-injection consistent with a phased approach to be completed by the end of 2020. During the first year (Phase 1), a comprehensive community and facility-based approach, including community health workers, known as health surveillance assistants (HSAs), and clinic-based providers, was implemented in seven districts to integrate DMPA-SC and DMPA-SC for self-injection into the public-sector FP program. It was also integrated into the services of 200 private sector clinics and in pharmacies in urban areas of four districts. This study took place in a Phase 1 district, Mangochi district, where DMPA-SC and self-injection scale-up was supported by two local organizations, University of Malawi-Polytechnic (UM-Polytechnic) and Youth Net and Counseling (YONECO).

### Sample selection

In both studies, clients were eligible to be interviewed if they met the following criteria according to the FP register data: 15 years of age or older, trained in self-injection, given DMPA-SC unit(s) to take home, and willing to provide written informed consent/assent. The Uganda eligibility criteria also specified that the client had been trained in one of the 12 sub-counties during Phase 1 or Phase 2 and given at least one unit to take home. For Malawi, the criteria specified that the client had been given at least two DMPA-SC units to take home at least 3 months prior to sample selection. We used de-identified client FP register datasets shared by WellShare in Uganda and UM-Polytechnic in Malawi to select potential participants within prespecified strata.

For the Uganda study, we selected equal numbers of potential participants from Phase 1 (*n* = 25) and Phase 2 (*n* = 25) sites in Iganga and Bugweri districts. We also purposively selected participants based on age, whether they had been trained by a VHT or clinic-based provider, and sub-county to ensure representation from different regions. We prioritized clients who had been trained a longer time before the interview and given more units of DMPA-SC and those who were considered covert users (this information was only available from Phase 2 clients’ FP registers). We defined a covert user as hiding their FP use from a partner, parent, and/or other person in power, such as their guardian or an older relative. WellShare staff contacted potential participants to invite them to participate using approved recruitment scripts. Of the 50 originally selected clients, 11 were replaced (e.g., because they were unreachable or unavailable for an interview), and two originally selected clients who were thought to be unavailable were replaced; however, later these two clients were interviewed in addition to their replacements.

For the Malawi study, we selected potentially eligible clients in Mangochi district based on age and whether they were trained by an HSA or clinic-based provider. Similar to Uganda, we prioritized clients who had been trained a longer time before the interview and given more units of DMPA-SC. Unlike in Uganda, we were not able to select covert users, as the FP registers did not collect this information. Study staff worked with HSAs and clinic-based providers to contact potential participants to confirm eligibility and invite them to participate using approved recruitment scripts. Out of the originally selected 60 clients, 33 (21 of whom were adolescents) were replaced (e.g., because they were unreachable, ineligible, or refused to participate due to being covert FP users). One originally selected client initially declined to participate and so was replaced; however, she later agreed to participate in an interview, and both she and her replacement were interviewed.

### Data collection

In March 2019, trained interviewers conducted interviews in Uganda using a pre-tested semi-structured interview guide with 50 clients who had been trained in self-injection of DMPA-SC. The client interview guide included questions on basic demographic information, when they had been trained to self-inject and by whom, how many times they had self-injected, what the health worker told them about how to dispose of used units, where they stored and disposed of used units, their preferences for storage and disposal, and whether they had heard of anyone getting pricked by a used unit.

In Malawi, from July–September 2019, trained interviewers conducted interviews using a guide similar to the one used in Uganda, which had been pre-tested in Malawi, with 60 clients who had been trained to self-inject DMPA-SC.

### Data analysis

All but one participant (in Uganda) agreed to have their interviews audio recorded. The interviews were transcribed verbatim and translated from Lusoga (Uganda) or Chichewa or Yao (Malawi) into English. The interviewer and note taker took detailed notes during the interview that was not recorded and then translated the notes into English for analysis.

The transcript data were coded using NVivo qualitative data analysis software (QSR International Pty Ltd. Version 12, 2018). For the Uganda study, two analysts conducted a thematic analysis using a codebook based on the interview guide, as well as an inductive, data-driven approach whereby emergent codes were applied to the raw data. For the Malawi study, three analysts used a similar process. For both studies, intercoder agreement was assessed on 10% of all transcripts until high consistency was achieved. Discrepancies were resolved through discussion, and adjustments made to the codebook and coding, as necessary. Data summary reports were generated, and matrices were used to identify and summarize key themes. Data were analyzed separately for adolescent and adult users, as well as covert and overt users, where possible, and the findings were compared, and differences noted when reporting the results.

### Ethical considerations

Both studies were reviewed and approved by FHI 360’s Protection of Human Subjects Committee. The Uganda study was also reviewed and approved by The AIDS Support Organisation Research Ethics Committee and Uganda National Council for Science and Technology, and the Malawi study was reviewed and approved by the National Health Sciences and Research Committee. For both studies, all study staff completed training on research ethics, the protocol, and informed consent administration. Study participants 18 years or older provided written informed consent, and participants ages 15–17 years provided written informed assent. We were granted a waiver for parental consent in both studies to protect the confidentiality of minors who were using FP without the knowledge of their parents. Participants were compensated 20,000 shillings in Uganda (about US$5) and 2300 Kwacha (about US$3) in Malawi for participating in an interview.

## Results

We interviewed 50 clients who were trained to self-inject DMPA-SC in Uganda and 60 in Malawi. Just under half of the samples in Uganda (*n* = 23) and Malawi (*n* = 28) were adolescents ages 15–19 years (Table 1). The mean ages of the samples were similar: 24 years in Uganda and 23 years in Malawi. Almost three-fourths of Malawi participants were married, whereas in Uganda, slightly more than half were married. All except one Malawian participant had at least one child, and most had two children. Fewer Uganda participants had children, with almost two-thirds having at least one child and the mean being two children. More than two-thirds of participants in Uganda had completed some secondary school, whereas most participants in Malawi had completed some primary education.

**Table 1:** Sociodemographic characteristics of study participants in Uganda (2019) and Malawi (2019)

	Uganda (*N* = 50) n (%)	Malawi (*N* = 60) n (%)
Age		
Mean age (range)	24 years (15–41)	23 years (15–39)
Adolescents (age 15–19 years)	23 (46)	28 (47)
Adults (age 20–41 years)	27 (54)	32 (53)
Current marital status		
Married	26 (52)	43 (72)
Not married	24 (48)	17 (28)
Has children	31 (62)	59 (98)
Mean number of children among those with children (range)	2 children (1–9)	2 children (1–6)^a^
Education level		(*n* = 58)
Never attend school	0 (0)	1 (2)
Some primary	14 (28)	46 (79)
Completed primary	2 (4)	1 (2)
Some secondary	29 (58)	9 (16)
Completed secondary	5 (10)	1 (2)

^a^One participant not asked this question

As there may be differences between adolescents and adults, we further examined whether adolescents were married and/or had begun childbearing, and Table 2 shows FP and self-injection characteristics by age group. Out of 23 adolescents in the Uganda sample, two were married and four, including the two who were married, had children (data not shown). In Malawi, out of 28 adolescents, all but one unmarried adolescent had children and 17 were married (data not shown). Most participants in both settings had used FP prior to being trained in DMPA-SC self-injection (Table 2). In Malawi, more than three-quarters of adolescents and almost all adults had previously used FP. However, in Uganda, adolescents were less experienced FP users compared to adults. The most commonly reported FP method that had previously been used was the intramuscular form of DMPA (DMPA-IM) in both samples. More than half of the Malawi participants and only slightly more than a quarter of Uganda participants were seeking DMPA-IM the day they were trained to self-inject (data not shown). In Uganda, where DMPA-SC administered by health workers had been available for some time, seven participants (two adolescents, five adults) reported receiving DMPA-SC administered by a health worker before being trained in self-injection. One Malawi participant had previously self-injected DMPA-SC because she was a participant in a randomized controlled trial on self-injection conducted from 2015 to 2017. More of the Malawi sample (82%) was trained to self-inject by CHWs compared to Uganda (68%).

**Table 2:** Family planning experience among study participants in Uganda (2019) and Malawi (2019)

	Uganda			Malawi		
	Adolescents *n* = 23 n (%)	Adults *n* = 27 n (%)	Total *N* = 50 n (%)	Adolescents *n* = 28 n (%)	Adults *n* = 32 n (%)	Total *N* = 60 n (%)
Used FP prior to being trained in DMPA-SC self-injection	12 (52)	25 (93)	37 (74)	22 (79)	29 (91)	51 (85)
Previous use of DMPA						
DMPA-IM	3 (13)	18 (67)	21 (42)	18 (64)	28 (88)	46 (77)
Health worker administered DMPA-SC	2 (9)	5 (19)	7 (14)	0 (0)	0 (0)	0 (0)
Other FP use prior to self-injection training^a, b^						
No method	11	2	13	6	3	9
Oral contraceptives	1	7	8	0	5	5
Implant	0	6	6	1	5	6
Intrauterine device (IUD)	0	2	2	0	1	1
Condoms	8	8	16	5	4	9
Other methods (withdrawal, standard days methods, lactational amenorrhea, other traditional)	4	3	7	0	1	1
Trained to self-inject by:						
Community health worker	20 (87)	14 (52)	34 (68)	23 (82)	26 (81)	49 (82)
Clinic-based provider	3 (13)	13 (48)	16 (32)	5 (18)	6 (19)	11 (18)
Has used DMPA-SC covertly	19 (83)	10 (37)	29 (58)	2 (7)	4 (13)	6 (10)

^a^Participants could have listed more than one method

^b^Because use of other FP methods was not systematically asked of all participants, percentages are not displayed

The largest difference between the samples was the number of covert FP users. There were only six covert users interviewed in Malawi compared to over half the sample in Uganda (*n* = 29; 19 adolescents, 10 adults). In Uganda, almost all adolescent users were also covert users (19 out of 23).

### Number of re-injections and units provided for future use

In order to provide context for storage and disposal, it was important to know how many units participants had taken home and self-injected prior to the interview. Most participants in Uganda reported having self-injected twice since being trained, and some had self-injected three or more times. Most participants in Malawi reported having self-injected once since being trained, and some had self-injected twice. Most participants in Uganda said they were given two to three units to take home, and most in Malawi reported being given three units to take home after being trained.

### Health workers’ disposal instructions

Almost all participants in both settings said the health worker discussed disposal with them during their self-injection training. They most commonly reported that health workers told them to return the units to the health worker (Uganda *n* = 33: 13 adolescents, 20 adults; Malawi *n* = 35: 17 adolescents, 18 adults). Most health workers told them to store the units in a plastic container prior to returning them to the health worker. Participants in both settings also specified that health workers told them to return the units to the health worker when the self-injectors had used them all.

About a quarter of participants in Uganda (mostly adolescents) and Malawi who said the health worker discussed disposal with them said they were told they could put used units into the pit latrine. Some in both settings said they were told that they could either return them to the health worker or put them in the latrine. In some cases, health workers’ instructions appeared to be intended to help the client do what worked best for the client’s situation. For example, an adult covert user in Uganda said, “*She told me that if I find time, I should bring them back. I should not dispose of them in the dust bin because my husband might see them, or if we burn* [the trash] *he could also find them. So if I fail to bring them back to her, then I should dispose of them in the latrine.”* Similarly, an adult participant in Malawi said, *“Bringing the used units, it’s not feasible because I am afraid that my children might play around with the used units. So I asked if it’s fine to dispose of them in the latrine to avoid my children being hurt … He said they dispose used units in a carton and throw in a bin, but at home we can dispose of them in a toilet since we don’t have bins, to avoid children getting hurt.”* A few participants in both settings said the health worker told them they could either put them in the latrine or bury or burn the used units. A few said health workers did not tell them how to dispose of the used units, but rather told them to keep and store the units. Most of these participants said they would ask the health worker how to dispose of them when they had used all their units. Three participants in Uganda and one in Malawi forgot what the health worker said.

### Storage and disposal methods

Self-injectors’ ultimate disposal method influences and is influenced by how they store the units prior to disposal. For example, if a self-injector intends to return used units to a provider then she must store them for some amount of time, whereas a self-injector who disposes in the latrine is likely to have a much shorter storage time. About two-thirds of Uganda participants (*n* = 34) and just over one-third of Malawi participants (*n* = 23) said they had disposed of at least one unit by the time of the interview (Table 3). In Uganda, the most common disposal method was returning used units to a health worker followed by a latrine. Of those in Malawi who had disposed of a unit, over half disposed in a latrine, while less than half returned units to a health worker.

**Table 3:** Reported disposal methods in Uganda and Malawi, among those who had disposed of at least one unit by the time of the interview

	Returned to health worker	Latrine	Other
**Uganda (*n*** **= 34)**			
Adolescents^a^	7	7	1 (sharps box at work)
Adults	15	5	1 (neighbor’s house)
Total^a^	22	12	2
**Malawi (*n*** **= 23)**			
Adolescents	6	8	0
Adults	3	5	1 (buried in rubbish pit)
Total	9	13	1

^a^Two adolescents disposed units in multiple ways (e.g., returned 1 to health worker and disposed 1 in latrine)

Among those who had not yet disposed of a unit, most were waiting until they were finished with all their units and planned to return them to a health worker. In Malawi, adolescent and adult disposal practices were similar in that both groups disposed used units into a latrine or returned them to a health worker, and generally disposed in a way they were told they should dispose. However, half of the adolescents and only a quarter of the adults had disposed of a unit by the time of the interview indicating that adolescents may dispose of the units more quickly than adults (e.g., disposing right away into the latrine rather than holding on to units until they returned to the health worker). This was different in Uganda, where more adults than adolescents had disposed of a unit by the time of the interview, but in this case most adults had returned units to health workers.

Among those who stored used units prior to disposal, most Uganda participants reported storing used units in a plastic container, specifically an empty Vaseline, lotion, or tablet container. A few stored used units in a hair product container, one in a box, and another in a bag. Several said they received these containers from health workers. Most specified that they put the container in a suitcase, under their bed, or somewhere in their house.

Most participants in Malawi reported storing them in a plastic container they obtained themselves (e.g., an empty Frozy drink bottle, lotion or Vaseline container, cooking oil bottle, etc.) in a bag in their bedrooms or “up high” (e.g., under the roof). Some participants did not use a container but stored used units in a plastic bag in another type of bag (e.g., a handbag or suitcase), with two acknowledging this was not a “proper” way to store them.

### Reasons for storage and disposal methods

Participants in both settings reported compliance with how the health worker instructed them to dispose of the units as the primary reason why they disposed of units in that way. For example, an adult in Malawi said, “*I was told to bring them back to the HSA, so I thought that was the rule about them and I didn’t want to be against it.”* As described above, some participants in both settings said they were told they could dispose of units into the latrine and often did so. For example, a covert user adolescent in Uganda who disposed of used units in the latrine to hide her use of FP said, “[The provider] *only told me to dispose of them in a pit latrine.”* One adolescent in Malawi described how she would have returned used units to the HSA, but since the HSA did not tell her to, she chose to dispose of them into the latrine, “*He could have told me to bring the used units to the HSAs place, but because he didn’t say to, that is why I disposed of them in a latrine.”*

The most common reason participants in both settings returned used units to health workers was because health workers told them to do so. Another common reason was that they felt they had to do this in order to get new units from the health worker. Further, some participants described the health worker as requiring confirmation of adherence through visual inspection of the used units as a condition of re-supply. For example, an adult participant in Uganda said, “*I have to take* [used units] *there as proof, and to explain that I have been using the medicine in order to be able to get more medicine.”* An adult in Malawi said, *“They said we should bring* [them back] *so that they should check if we really self-injected or not. I also think it’s a good thing that the HSA should be checking to see if I really self-injected or not*.”

The main reasons participants disposed of units in the latrine differed by setting. In Malawi, the primary reason was to keep used units away from children and other people so they would not get hurt from needlesticks, although one adult said she had put it into the latrine to hide it from her husband. For most who disposed of the used units in a latrine, they reported that the health worker had told them they could do so, while others said the health worker did not discuss disposal at all. One Malawi adult participant said she had been told to store the units in a bottle but chose to dispose of the used units in the latrine*: “Ahh they* [HSAs] *explained that we should find a small bottle with a lid on top and* [put them] *in there. However, I did not use this method. After injecting myself I threw it in the toilet … That’s where a child cannot pick it up and start to play with it … The bottle would also be a good place. However, it would make us busy to carefully keep* [used units] *in the house, once you have put it in the bottle anyhow, or outside, children will take it and will start to do what, to play with it.”*

Similar to participants in Malawi, one of the main reasons participants in Uganda disposed of used units in the latrine was to keep them away from children and others; however, more participants in Uganda said they felt that disposing of used units in the latrine was more convenient and more discreet than returning them to a health worker. For example, when asked why she disposed of used units in the latrine, one covert adolescent user said, “*I don’t want the bottle to be seen there, for everybody to start wondering what this is used unit for and where it came from*.” Almost all participants who disposed of used units in the latrine were told they could. Most participants who said providers told them they could dispose of the units in the latrine were adolescent covert users, and two specifically said they were told to dispose of the used units in the latrine because they were covert users. For example, “*For those who live with their tough parents, we were advised to immediately put the used injection units in a tin after we get done to self-inject and dispose of them in a pit latrine when the right time of disposing them came.”*

### Preferences regarding storage and disposal

The majority who were asked in both Uganda and Malawi (42/47 in Uganda and 45/56 in Malawi) said they would prefer to dispose of used units in a latrine primarily because they worried that storing used units could result in needle pricks, and because it was more convenient than returning units to a health worker. Even though most women wanted to dispose of them in the latrine, several specifically discussed not doing this because the health worker had told them not to do so. For example, an adult overt user from Uganda who returned units to the health worker said: “*The right place is in the latrine… No one will ever reach there, and there is no way that it will prick anybody … If the provider told me to dispose of them in the latrine, I would have done it. But she told us to bring the used units back and she disposes of them.”*

Privacy was a significant concern for covert users in both settings. Oftentimes this was expressed when covert users were describing the steps they took to conceal new units prior to injection and continued through their discussion of disposal of used units. For example, more than half of adolescent covert users in Uganda explicitly mentioned privacy concerns related to storing and/or disposing of units, as illustrated by this quote, “*I see it’s difficult* [to return the units to the health worker]*, because I will be escaping all the time and yet am afraid of my parents finding out and by the time they find out I will be punished.”* Three (two adults and one adolescent) covert users in Uganda and two (adults) in Malawi reported having either new or used units discovered by a partner or family member. Most reported that they did not experience any problem after the discovery, however, one Uganda adult covert user said her husband threw her unused units in the latrine after finding them. An additional adolescent covert user in Malawi said she planned to stop self-injecting because she did not want her husband to find her units.

### Experiences with needlesticks during storage and disposal

Participants’ concerns around the potential for needlesticks were prevalent in both settings. During self-injection training, several participants mentioned that the health worker said they should store and dispose of used units in a certain way (i.e., in a puncture-proof container) so as not to risk anyone pricking themselves. Most participants expressed concern that children could access and prick themselves with the used units if they found them. As described above, this was also one of the primary reasons disposal in the latrine was preferred. All participants who were asked if they or anyone they knew had ever been pricked by a used unit (*n* = 50 in Uganda; *n* = 58 in Malawi) said no.

## Discussion

Waste management is an important priority as countries introduce self-injection of DMPA-SC. Therefore, we conducted qualitative studies in Uganda and Malawi, which had recently introduced DMPA-SC self-injection, to explore self-injectors’ experiences disposing used units after injection in order to inform programs. We prioritized interviewing adolescent and covert self-injectors to determine if current recommendations for waste disposal—storing used units in a container until they can be returned to a health worker—were feasible for these users.

Returning units to health workers was not preferred by most self-injectors in either setting. Most participants reported disposing of the used units as they were instructed by the health workers who had trained them to self-inject. However, about one-quarter of participants reported that the health worker had instructed them to dispose of the used units in latrines, though this is not the current recommendation in Uganda and Malawi. In some cases, health workers’ instructions appeared to be tailored to the clients’ personal situation such as being a covert FP user, suggesting that some health workers may not feel that the current recommendations are feasible for every user.

Even though most self-injectors wanted to dispose of the used units in the latrine, several specifically discussed not doing so because the health worker had told them not to, highlighting the importance of clear and accurate messaging during self-injection training. The current recommendation in Uganda and Malawi is that clients store units and then return them to a health worker at the client’s convenience (e.g., so that clients do not have to return them to the health worker after each injection, which would defeat the primary advantage of self-injection). However, we found that the message about the timing of the return being at the clients’ convenience may be getting lost. In addition, some participants reported the perception that they must keep the used units for health workers’ visual inspection as a verification step in order to receive future doses of DMPA-SC.

Our findings that some adolescent covert users in Uganda felt that storing units and returning them to health workers was challenging due to privacy concerns and more adolescents in Malawi disposed of used DMPA-SC units sooner than adults suggest that returning units to the health worker may not be feasible or may reduce the attractiveness of self-injection for populations who may benefit most from self-injection [12, 13], such as adolescent and covert users. Compared to immediate disposal into the latrine, returning used units to health workers means that self-injectors in the settings we studied must store units for a longer period of time, increasing the likelihood that they will be discovered by someone who may not approve of their use of FP. Self-injectors are already having to store their new units prior to injection, and being able to dispose of used units more quickly may make self-injection more viable for covert use. This is particularly relevant to adolescents who face challenges to accessing contraceptives in general [31–34] and value privacy and discreetness in contraceptive use [3–5]. It should be noted, however, factors such as the client’s geographic proximity to the health worker and cost of transportation may influence how easy it is for self-injectors to return the units, and these factors should be considered when planning self-injection programs.

Participants in Uganda most often returned used units to health workers, whereas in Malawi, slightly more had disposed into the latrine. However, this finding may be influenced by the timing of the interviews we conducted in each setting, as participants in Uganda typically had been self-injecting for longer compared to participants in Malawi and therefore had more used units and more time to dispose. Further, most Malawi participants who had not yet disposed of used units planned to return them to health workers. Our findings may also be influenced by self-injection being a new practice in both countries. Specifically, this could affect how women dispose of or report plans to dispose of used units—perhaps as they hear about more women disposing into the latrine and discover that they do not have to bring back used units to the health worker in order to get new units, more self-injectors will ultimately dispose of used units in the latrine. Or, perhaps as the method is available for longer, health workers may receive additional training and supervision that encourages them to more strongly instruct women to return units to them and this may result in more units being returned to health workers. It is important to note that we recruited for the Malawi study in the same district where we conducted a randomized controlled trial comparing continuation of DMPA-SC administered by health workers to self-injection in 2015–2017 [14], which could also influence our findings. During the trial, research participants were instructed to dispose of used units in latrines. However, the Malawi MOH adopted recommendations to return used units to a health worker, in accordance with its guidelines on infection prevention and waste management, for the national scale-up which began in 2018, so it may take some time for the new guidance to be fully adopted.

Another factor that could influence disposal is whether adolescents are married or have begun childbearing—conditions which could decrease stigma for adolescents accessing FP services and, by extension, could make returning units to health workers easier. When we examined the breakdown of adolescents in these two categories, we observed that our two study samples were very different. In Uganda very few adolescents (4 out of 23) were married and/or had begun childbearing, whereas in Malawi almost all adolescents (27 out of 28) had these experiences. In addition to affecting disposal methods, this sample-level difference likely explains the reason why many more Ugandan participants were covert FP users compared to the Malawi sample.

Self-injectors said they preferred to dispose of used units in the latrine primarily because they worried that storing used units prior to returning them to a health worker could result in needlestick injuries to children and others. However, no study participants in Uganda or Malawi reported needlestick injuries to themselves or others. We are not aware of any other study that has specifically asked about the occurrence of needlestick injuries associated with DMPA-SC self-injection to be able to determine if our qualitative study findings are typical. Our scan of the diabetes literature for related insights indicated that even though diabetic patients have been injecting insulin in home settings for a long time, at-home disposal practices among this population are understudied [35, 36]. However, our search found that, particularly in the absence of instruction, diabetics dispose of their sharps in the most convenient location, consistent with our findings, but in this case, diabetics usually disposed in the household garbage [35, 36].

### Limitations

While we reached data saturation for the primary objective, which was to describe the waste disposal practices of samples of self-injectors in Uganda and Malawi, our study has several limitations. In both countries, we aimed to select equal numbers of younger adolescents (ages 15–17), and adults (age 18 and older) because younger adolescents are an understudied group. However, we had difficulty recruiting younger adolescents and therefore expanded the age range of adolescent participants to 19 years, consistent with a common global health definition of older adolescents [1, 2, 37], to reach our desired sample sizes. It was difficult to recruit 15–17-year-old self-injectors in Uganda because there were not many self-injectors in that age range and, in some cases, they were not traceable or were in a different location for school. In Malawi, recruitment of 15–17-year-old self-injectors was difficult mainly because they were not traceable. Also, in Malawi, the HSAs who helped recruit clients for the interviews in some cases seemed to act as gatekeepers and would not recruit 15–17-year-olds specifically—they often cited that these were “schoolgirls” who were using DMPA-SC covertly and would not want to do an interview. Additionally, there were sometimes discrepancies between self-reported age and FP register age in both countries, which affected our ability to recruit equal numbers of adolescents and adults.

We had hoped to interview more covert users for the study. In our Malawi sample, only six reported that they were either currently hiding their use of DMPA-SC or had previously hidden their use of it and were no longer hiding it. In Uganda, the FP registers (in Phase 2) we used for recruitment indicated whether the client was using DMPA-SC covertly, whereas the FP registers in Malawi did not include this information. In addition, almost all of the adolescents we interviewed in Uganda were covert users, indicating that covert use may be high among adolescents; thus, it is possible the mostly overt adolescent users we interviewed in Malawi were not representative of most adolescent self-injectors and this may also be because the two adolescent samples differed on key characteristics as discussed above. Since we did not reach our desired sample sizes for 15–17-year-olds in either site or covert users in Malawi we do not believe we reached data saturation for these groups. We recommend that future research explore the experiences of self-injectors from these groups to further validate our findings.

Finally, as with all qualitative data, we relied on self-report; therefore, recall and social desirability biases may have influenced participants’ responses. Indeed, many participants in both settings had trouble recalling training and re-injection dates during the interview, making it difficult to determine exactly how long they had been using DMPA-SC. The use of qualitative methods also meant we were not able to quantify the disposal of each unit dispensed to the participants. Future quantitative studies with a complete accounting of each participant’s used units would be beneficial to further validate our findings.

We attempted to reduce recall bias by interviewing current self-injectors after they would have self-injected and disposed of at least one unit. However, we needed to balance the risk of recall bias alongside the risk of information bias because self-injectors could dispose of a used unit right after injecting at three months post-training or they could store multiple used units for up to a year prior to disposal. We attempted to reduce social desirability bias by hiring interviewers who were not family planning providers and, whenever possible, we employed female interviewers to match the gender of the participants. Further, all interviewers attended a five-day training that included role-playing exercises focused on rapport building and non-judgmental interviewing techniques that culminated in each interviewer conducting a pre-test interview and receiving individual feedback on their interviewing skills.

### Strengths

Our findings are similar to and contribute to the growing literature about DMPA-SC disposal [38]. Limited published literature exists related to storage and disposal of DMPA-SC, and almost all has been in the context of self-injection offered within research contexts, including Malawi [19, 22], Senegal [39], Uganda [30], and the Democratic Republic of the Congo [40]. This is the first study to report on the waste management experiences of adolescent and covert self-injectors within the context of program scale up—important populations expected to especially benefit from self-injection. Despite the challenges of recruiting adolescents, we were able to interview a sizeable number of 15–17-year-old self-injectors (13 in Uganda and 11 in Malawi), which offers valuable information on this understudied age group. Another noteworthy strength of our study was that in Uganda, we were able to interview 29 covert users, most of whom were adolescents.

### Recommendations

Based on our study findings, and in line with others’ recommendations related to self-injection [41], we make a number of recommendations for FP programs that integrate self-injected DMPA-SC:

1. We recommend ensuring clear messaging about the correct storage of used units, as well as the process and timing of disposal. We observed a strong desire among participants to be compliant with the health workers’ instructions. Training should emphasize that the timing to return used units to the health worker is entirely dependent on the convenience of the self-injecting client.
2. We recommend against countries requiring visual inspection of used units as a prerequisite for re-supplying self-injectors. There could be a variety of reasons, including safety, why self-injectors may not keep their used units, and such a requirement could increase barriers to access to self-injection.
3. For overt users, we recommend helping clients identify appropriate containers or providing containers, along with safe handling instructions on transferring waste to the health system, to reduce the risk of needlestick injuries. Our findings suggest that most self-injectors, *especially those who are overt FP users*, could successfully return units to the health worker if we could assuage their concerns about needlestick injuries associated with this disposal method. This is supported by a study in Uganda which found DMPA-SC self-injection, including the cost of providing a disposal container to clients, to be cost-effective compared to DMPA-IM administered by health workers [42], however this may not be true in other contexts. Providing containers that reduce needlestick risk could address overt self-injection users’ concerns, while at the same time helping them to meet current local disposal guidelines. In addition, self-injectors and the health workers who assist them need clear guidance on how to safely transfer used units from clients’ containers to their medical sharps waste containers [29].
4. For covert users, we recommend that more convenient disposal solutions be identified so that self-injection can reach its maximum impact as a convenient contraceptive method. The potential risk and stress of disclosure that storing used units places on covert and adolescent users may discourage them from using an FP method they might otherwise use. In addition, most self-injectors do not find returning used DMPA-SC units to health workers convenient. Future research could explore engaging the private sector in disposal practices to determine if placing sharps boxes at pharmacies, drug shops or other locations throughout communities makes safe disposal more convenient for self-injectors.

## Conclusions

As of October 2019, DMPA-SC is registered for self-injection in more than 50 countries worldwide, including Uganda and Malawi [43]. Furthermore, the World Health Organization (WHO) has recently added self-injection as a “strong recommendation” to their consolidated guidelines on self-care interventions for health, which may accelerate the availability of self-injection worldwide [44]. Self-care interventions, which reduce the need to travel to or have contact with health facilities and workers, are particularly relevant during epidemics (e.g., Ebola) or pandemics (e.g., COVID-19). Solutions for household medical waste disposal will likely be increasingly important as more self-care interventions are developed and brought to scale. However, as the WHO notes, we must take special care when planning and implementing self-care interventions to ensure equity [44]. By shifting responsibility to individuals, we need to equip all individuals with the tools to appropriately use self-care interventions. Related to DMPA-SC self-injection, some groups may not even try self-injection if they cannot safely and easily dispose of used units. Furthermore, if the required disposal process exposes adolescent and covert FP users’ FP use to disapproving partners or family members, this could result in harm. Our findings are timely as countries plan for and implement self-injection programs and develop disposal guidelines that guarantee the safe handling of medical waste, while ensuring that self-injection can realize its maximum impact and be accessible to all who could benefit from this innovative contraceptive option, especially adolescent and covert FP users.

## Data Availability

The data analyzed during the current study are available from the corresponding author on reasonable request.

## Abbreviations

APC: Advancing Partners & Communities project
CHW: Community Health Worker
CIFF: Children’s Investment Fund Foundation
DMPA: Depot medroxyprogesterone acetate
DMPA-IM: Intra-muscular Depot medroxyprogesterone acetate
DMPA-SC: Depot medroxyprogesterone acetate delivered sub-cutaneously
FHI 360: Formerly Family Health International
FP: Family Planning
HSA: Health Surveillance Assistant
IRB: Institutional Review Board
IUD: Intrauterine device
mCPR: Modern contraceptive prevalence rate
MOH: Ministry of Health
UM-Polytechnic: University of Malawi-Polytechnc
USAID: United Stated Agency for International Development
VHT: Village Health Team
WHO: World Health Organization
YONECO: Youth Net and Counseling
